# Where will the money come from? Alternative mechanisms to HIV donor funding

**DOI:** 10.1186/1471-2458-14-956

**Published:** 2014-09-16

**Authors:** Itamar Katz, Subrata Routh, Ricardo Bitran, Alexandra Hulme, Carlos Avila

**Affiliations:** Abt Associates, 4550 Montgomery Ave, Suite 800 North, Bethesda, MD 20814 USA; Bitrán & Asociados, Avenida Presidente Riesco N°s 5711, Office 802, Las Condes, Santiago, Región Metropolitana 756 1114 Chile

## Abstract

**Background:**

Donor funding for HIV programs has flattened out in recent years, which limits the ability of HIV programs worldwide to achieve universal access and sustain current progress. This study examines alternative mechanisms for resource mobilization.

**Methods:**

Potential non-donor funding sources for national HIV responses in low- and middle-income countries were explored through literature review and Global Fund documentation, including data from 17 countries. We identified the source, financing agent, magnitude of resources, frequency of availability, as well as enabling and risk factors.

**Results:**

Four non-donor funding sources for HIV programs were identified: earmarked levy for HIV from country budgets; risk-pooling schemes such as health insurance; debt conversion, in which the creditor country reduces the debt of the debtor country and allocates at least a part of that reduction to health; and concessionary loans from international development banks, which unlike grants, must be repaid. The first two are recurring sources of funding, while the latter two are usually one-time sources, and, if very large, might negatively affect the debtor country’s economy. Insurance schemes in five African countries covered less than 6.1% of the HIV expenditure, while social health insurance in four Latin American countries covered 8–11% of the HIV expenditure; in Colombia and Chile, it covered 69% and 60%, respectively. Most low-income countries will find concessionary loans hard to repay, as their HIV programs cost 0.5–4% of GDP. Even in a middle-income country like India, a US$255 million concessionary loan to be repaid over 25 years provided only 7.8% of a 5-year HIV budget. Earmarked levies provided only 15% of the annual HIV funding needs in Zimbabwe and Kenya. Debt conversion provided the same share in Indonesia, but in Pakistan it was much higher - the equivalent of 45% of the annual cost of the national HIV program.

**Conclusions:**

Domestic sources of funding are important alternatives to consider and might be able to replace donor HIV funding in specific country contexts, coupled with effective prioritization and efficiency measures. Successful resource mobilization design and implementation require close collaboration with other sectors, particularly with the Ministry of Finance, to make sure that the new financing mechanism is fully synchronized with economic growth and that HIV investments yield returns in the form of higher social benefits.

## Background

In a vast majority of low- and middle-income countries, national health systems face financial sustainability challenges as donor funding declines. Donor dependence is particularly high for HIV programs in low- and middle-income countries where HIV prevalence is high. Combined donor assistance to HIV programs in 127 of these countries amounted to 49% of total HIV funding in 2011. In many low-income countries, and in some middle-income ones, the dependence is higher: in Cambodia, Niger, and Tajikistan, for example, more than 75% of HIV spending comes from outside agencies, with the government contributing less than 25% [1]. HIV programs tend to rely more on donor funding than does the wider health sector. In lower middle-income Ukraine in 2009, donor funding as a percentage of total health expenditure was only 0.3%, whereas it accounted for 46% of the funding for the national HIV program [2]. In upper middle-income Jamaica in 2008, donor assistance to the health sector overall amounted to 1.5%, as opposed to 51% for the country’s HIV program [3].

HIV has long-term implications for treatment costs because it is a chronic condition that requires medical attention throughout the life of the patient. In addition, as an infectious disease, it requires sustained resources for prevention. To execute the investment framework led by UNAIDS, a global HIV strategy budgeted at between US$16 billion and US$22 billion will be required annually between 2011 and 2020 to effectively fight the AIDS epidemic [4]; in 2011, US$16.8 billion was available globally [1]. After rapidly expanding, HIV funding from donor countries has flattened out between 2008 and 2012, ranging between US$6.9 and US$7.9 billion per year [5]. The recent global recession, coupled with increasing competing demands for new causes such as non-communicable diseases, climate change, and the environment, might further jeopardize increased donor funding for HIV. The Global Fund to Fight AIDS, Tuberculosis and Malaria, PEPFAR, and other donors have already begun to consolidate and focus their funding on certain priorities and are demanding greater counterpart participation. A key goal of the five-year PEPFAR reauthorization in 2008 has been to transition from emergency response to country-led sustainable HIV programs [6]. To boost country contributions by recipient governments, in 2011 the Global Fund issued eligibility and counterpart financing guidelines, requiring countries to match the grant funds with a contribution based on their income level [7]. For example, low-income countries are required to match only 5% of their Global Fund financing, while upper middle-income countries are required to match 60%. In parallel, a number of countries receiving donor assistance for their HIV programs have graduated in recent years to upper or lower middle-income groups as a result of the economic growth, enabling them to increase their share in funding of their HIV response. Seventeen of them are now ineligible for Global Fund financing.

The above trends require policymakers of national health sectors to expand their fiscal space to address the financial sustainability of the HIV programs as their donor funding is set to decline. A long-term financial sustainability plan should include cost reduction, improved allocation of funding (both of tax money and within health programs), and resource mobilization [8–13]. The remainder of this paper describes accessing and/or creating alternative resources to donor funding, alternatives that can be proposed by policymakers of health programs.

## Methods

The paper reviews resource mobilization alternatives that have been deployed or are being considered by national HIV programs in low- and middle-income countries (including countries such as Chile and Uruguay, which recently became high income). The alternative financing mechanisms presented here derive from consultation with health financing experts and review of both academic and grey literature. In addition to literature known to the authors, a search in PubMed and Google Scholar was conducted, including using various combinations of HIV and/or AIDS with funding, tax, levy, earmarked, concessionary, loans, deb conversation, Multi-Country HIV/AIDS Program (MAP), risk-pooling, insurance, social health insurance, and resource mobilization. The criteria for inclusion were as follows:

1. Funding mechanisms that policymakers of health programs are usually in a position to promote. We did not include options such as improving tax revenue administration and printing money. While those will expand the fiscal space, they might or might not be directed to the health sector.
2. Funding mechanisms that channel the funds to the country executing the mechanism. As such, we excluded global initiatives such as UNITAID, a global health initiative financed mainly by a solidarity levy on airline tickets, and the International Finance Facility (IFF), which issues bonds on global capital markets against the security of government guarantees. This increases funding for global health in the short term [14].
3. Funding mechanisms that have been examined by HIV programs in low- and middle-income countries. This criterion eliminated options such as borrowing money in general financial markets (where interest rates tend to be high) and social impact bonds, where the government contracts out provision of social services to a private sector company that is paid based on its reaching pre-defined targets, with no upfront payment [15].
4. Options that do not require substantial support from additional resources to cover the antiretroviral treatment (ART) cost, such as community-based health financing, which similar to health insurance provides health coverage, yet the funding generated is sufficient only for basic health services, not for HIV-related biomedical services.

**Figure 1:**
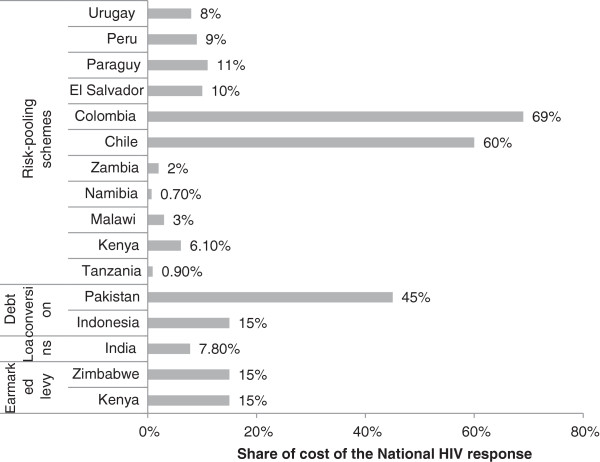
**Magnitude of strategies that countries are using to finance HIV programs.** The examples of risk-pooling schemes from Africa include HIV expenditures of both private firms and private health insurance, and as such the share of health insurance alone is lower.

Per funding source, we provide a brief explanation on the nature of the funding source, the financing agent [16], whether it is a recurring funding source or one-off, enabling and risk factors, and magnitude and/or feasibility of resources it mobilizes. We provide country examples on the extent to which a funding source covers the annual cost of the HIV response, the latter taken from funding requests made by the countries to the Global Fund. Table 1 and Figure 1 summarize the key features of the four strategies identified. This is followed by a case study of efforts of decreasing dependency on donor funding in Kenya.

**Table 1:** **Summary of potential strategies for additional financing of HIV programs**

Options	Financing sources	Financing agents	Recurring funding source	Examples of magnitude/feasibility
Earmarked levy or additional taxation for HIV	Domestic, private	Ministry of Finance	Yes	• Zimbabwe – 15% of the requested annual funding from the Global Fund for 2014–2016. • Kenya – 15% of the estimated cost of the 2012/13 national HIV response.
Concessionary loans for HIV programs	Domestic (when the loan is repaid)	Ministry of Finance	No	• India – A US$255 million concessionary loan to this middle-income country provides 7.8% of the 5-year HIV budget and is to be repaid over 25 years. • Low middle-income countries – Most will find concessionary loans hard to repay, as their HIV programs cost 0.5-4% of GDP.
Debt conversion	External	Ministry of Finance	No	• Indonesia – Debt conversion mobilized US$35.5 million for health programs, the equivalent of 15% of the funding needed for the national HIV response of US$241 million. • Pakistan – Debt conversion was the equivalent of 45% of the annual cost of the HIV program. However, HIV prevalence in Pakistan is very low (<0.1%), and with it the required financial resources.
Risk-pooling schemes and special social assistance programs covering HIV services	Domestic, public and private	Health insurance entities	Yes	• Social health insurance (SHI) schemes provide 60% of the HIV funding of Chile, and 69% in Colombia; the proportion is substantially lower in the following: El Salvador 10%, Paraguay 11%, Peru 9%, and Uruguay 8%. • Kenya, Malawi, Namibia, Tanzania, and Zambia – Private insurance is less than 6.1% of the national HIV expenditure. • Rwanda – In this low-income country, a health insurance scheme was financially supported by the Global Fund.

## Results

### Earmarked taxes

Earmarked taxes seek to increase government funds available for HIV and/or health more broadly by increasing tax revenue. A number of countries have introduced special taxes that are earmarked (i.e., exclusively allocated) for health interventions, for example, on air traffic and tobacco products [17].

Earmarked taxes have several pros and cons (Table 2) [18, 19]. The pros include a greater willingness of taxpayers to pay a tax with associated positive health benefits [20] and the ability to protect the funded program/s from general budgetary cutbacks and/or de-prioritization. Cons include loss of control over total expenditure, in particular when priorities of a country change. Earmarked taxes, where mismanaged, may lead to inefficiencies and misuse of funds.

**Table 2:** **Earmarked tax: pros and cons**

For	Against
• Households associate the benefits of the government expenditure with the tax paid and are more prepared to pay.	• Earmarking means a loss of control over total expenditure.
• Earmarking may provide a more consistent source of funds for expenditures that yield high benefits but may not be high on the political agenda, such as road maintenance.	• Earmarking circumvents the budgetary process and review and may distort and misallocate funds.
• Earmarking shields expenditures from the uncertainties of legislatures that may cut spending.	• Rights to earmarked revenues become entrenched with funding no longer based on agreed priorities.
	• Less transparency may lead to inefficiencies and misuse of funds.
	• Earmarking can facilitate attempts to create monopolies and abuse of monopoly power.
	• Earmarking could lead to cutbacks (or expansion) of services wholly unrelated to need.
	• Earmarking leads to less flexibility at the margin to reallocate funds when the budget is under stress.
	• Earmarking is incompatible with good cash management.

Sources: [18, 19].

If this strategy is to be pursued, designers must consider several issues when determining the item to tax and the amount of tax, including: its potential to generate sufficient revenues, the reduction in revenue if it causes a decrease in demand for the taxed good/service, the ease of implementation – for example, straightforward collection processes with low administrative (operating) costs – and the likelihood the tax will affect businesses. The ability to implement such tax depends on the current tax burden. In low-income countries, it can be expected that the tax share will be at least 15% of the GDP. In countries such as Tanzania, where the tax ratio is less than 13%, increasing the fiscal space is possible, yet where ratio already exceeds this rate, as in Zambia and Malawi, adding a new tax might be difficult [9]. Political will and consensus among the government and lawmakers is important in implementing special levy schemes. Finally, reliance on social security may limit the acceptability to the public of yet another employment tax to cover the costs of HIV services. Reliance on social security is common in most countries in Latin America.

Zimbabwe introduced an AIDS levy in 1998, and it became operational in January 2000, when a 3% earmarked (or special purpose) tax was imposed on employers and workers. Funds collected from this tax are channeled to the National AIDS Trust Fund. In 2011, the government collected US$26 million through the trust fund – a figure that is expected to rise to US$30 million in 2012 [21]. This funding source is substantial, yet still insufficient – the average annual funding requested from the Global Fund by Zimbabwe for 2014–2016 was US$195 million, more than six times the expected revenue from this tax in 2012. Nevertheless, this method has worked in this low-income country, where other innovative financing mechanisms described in this paper are harder to implement. As a tax on employment, this special earmarked tax for HIV increases the cost of labor and may inhibit formal employment. However, the obvious benefit of this tax is that it has collected a substantial amount of revenue that, if managed well, is put to good social use.

### Concessionary loans

Loans for health and HIV programs with favorable terms, such as low interest rates and deferred payment schedules, are available for eligible countries from sources such as the International Monetary Fund, World Bank, and regional development banks, and other financing institutions. While a loan is required to be repaid, unlike a grant, its selection and implementation mechanism focuses on the efficient use of the funds, inclusive of project finance supervision. A number of low- and middle-income countries use World Bank loans and grants to support their health sector programs. Many of them also used World Bank funds for implementing HIV projects in the last decade. In the African region alone, between 2001 and 2011, the World Bank provided US$2 billion to an estimated 50 countries and regional entities for HIV projects. Angola, Botswana, Ethiopia, Kenya, Malawi, Nigeria, Swaziland, and Tanzania are but a few that did so [22]. The World Bank has greatly scaled back such funding in recent years as both the Global Fund and PEPFAR started offering large-scale grants [23, 24], though there are still HIV programs being financed through it: in June 2013, India’s HIV national response received US$255 million in a loan with no interest, and ability to repay over a period of 25 years [25]. This represents 7.8% of the 5-year HIV budget [26].

In addition to loans from development banks, governments can pursue ODA loans, which have a grant element of at least 25% because of their low-interest and/or long-term payment schedules. These bilateral agreements between governments focus on program-level country ownership, while limiting the burden of the donor country. Japan and France are two countries that frequently give loans in a larger proportion than grants, providing US$7.5 billion and US$1.4 billion, respectively, in loans for a wide range of development projects in 2010 [27]. However, because of the availability of donor funding, concessionary loans for HIV are limited to date. This might change if demand from countries for such loans increases.

Low-interest loans issued by international development agencies typically have conditions that the host country does not like or cannot fulfill. Thus, securing such loans might involve a lengthy negotiation process between the recipient country and the lender. Repaying the loan may become difficult if the local currency depreciates against the currency in which the loan needs to be repaid, and/or if government tax revenue declines. Loans will be hard to repay in most low-income countries, where costs of HIV programs are 0.5–4% of GDP, substantial given that public health expenditures among African low-income countries averages only about 2.5% of GDP, and domestic government revenues are frequently below 15% of GDP [28]. But if the HIV project financed by the loan is well conceived and managed, it should yield returns in the form of higher social benefits (e.g., reduced incidence of HIV, reduced mortality and morbidity, lower work absenteeism, higher productivity); these may also translate into higher economic output and tax revenue in the longer term. Thus, the focus should be on the appropriate design and implementation of the activities to be financed through the loan.

### Debt conversion

Debt conversion for social investments began in 1996 with the launch of the Heavily Indebted Poor Countries (HIPC) Initiative that linked debt relief and poverty reduction. A prominent example targeting HIV, tuberculosis, and malaria programs is the Global Fund’s Debt2Health program, announced in 2007, which channels resources of developing countries with high debt and disease burdens away from debt repayments and toward investments in health [29].

Under Debt2Health, the Global Fund works with its partners to identify debt conversion opportunities, and then negotiates a three-party agreement between the bilateral or multilateral creditor, the debtor, and the Global Fund. According to the agreement, creditors forgo a portion of their claims on the condition that the beneficiary country (the debtor) invests an agreed counterpart amount in its national HIV, tuberculosis, and/or malaria programs, through an approved Global Fund grant. The funding provided through Debt2Health is disbursed by the Global Fund to the beneficiary country through the fund’s normal performance-based grant mechanism.

As of May 2013, four Debt2Health agreements plus one framework agreement have been signed. Germany and Australia are the creditor countries and Indonesia, Pakistan, and Côte d’Ivoire are the contracting beneficiaries. A total of €163.6 million has been committed to these Debt2Health agreements, with half of this amount – €81.8 million – paid to the Global Fund for investment in the beneficiary countries through the standard Global Fund processes and systems, and the other half unconditionally written off by the creditor countries.

Australia used the mechanism to execute a debt swap in Indonesia in 2010, canceling US$75 million of debt, with Indonesia investing US$35.5 million into health programs in the country. While a substantial amount, it provides only 15% of the average annual funding needed for the national HIV response of US$241 million from 2013 to 2015 [30, 31]. An agreement between Germany and Pakistan was signed in 2008, in which Germany cancelled €40 million and Pakistan began to invest €20 million in Global Fund-approved programs in the country. This amount translates into 45% of the annual cost of the HIV program in Pakistan between 2013 and 2015 (thought the Debt2Health funding could be used for fighting malaria and/or tuberculosis, the latter being a larger health issue in Pakistan compared to HIV and malaria). This is because, with an HIV prevalence of less than 0.1%, the cost of the HIV response in Pakistan is much lower than in many countries with generalized HIV epidemics [31, 32].

In addition to debt reduction and channeling public investment into social sectors, Debt2Health results in foreign exchange savings because the investments are in the local currency. Challenges include transaction costs because debt conversions can be complex to negotiate, execute, and monitor. Also, the amount to be paid to the Global Fund by the debtor country is usually due sooner than the original debt repayment, and the local Treasury may not have funds available to pay the Global Fund. Finally, the increased government spending could be inflationary [33]. Those challenges can be mitigated with solid planning and implementation of the debt conversion.

### Risk-pooling schemes and special social assistance programs

Health insurance is an organizational arrangement devised to offer financial protection to a large group of individuals (known as the *risk pool*) from the costs associated with preventing and treating ill health. Health insurance is based on the principle that the financial risk of a few individuals is spread among a large pool of mostly healthy individuals. If all contribute a periodic insurance premium, the insurer can collect and manage the premium revenue to finance the treatment costs of the few who become sick. In the absence of health insurance, people needing medical services, especially those struck by catastrophic conditions such as cancer, HIV, obstetrical emergency, or severe accidents would find it difficult or impossible to obtain the resources necessary to cover the cost of treatment [34]. In a recent study in Kenya, it was shown that among adult patients hospitalized in a public referral hospital, insurance coverage was associated with decreased in-hospital mortality [35].

Among private voluntary health insurance plans, several corporations fund HIV services for their employees and dependents and some of the private health insurance companies have begun to offer coverage for HIV services [36, 37]. However, given the high cost of HIV services, in particular of ART, which is taken for life, many of the public and private health insurance programs still do not cover HIV services altogether or are ambiguous regarding the coverage of these services. For example, Kenya’s National Hospital Insurance Fund does not cover outpatient services, including ART or outpatient treatment of opportunistic infection. Hospitals and other providers in Kenya must recover these costs from patients, government, and donors [38]. The limited use of health insurance for HIV-related services is shown in a recent review of health insurance plans in five African countries – Kenya, Malawi, Namibia, Tanzania, and Zambia – where private insurance covered less than 6.1% of the national HIV expenditure [39].

Several efforts in Africa to include HIV services in health insurance plans were supported by donor funding. There are two interesting experiences, which differ in terms of national income level and structure of the services. In Rwanda, a low-income country, the Global Fund provided support to enhance financial access to AIDS, tuberculosis, and malaria health-related services by subsidizing health insurance for the very poor. The strong leadership of the Ministry of Health enabled this Global Fund-funded project to dramatically improve the financial access of its target group, reaching approximately one Rwandan in six. Improved financial access went hand-in-hand with increasing health service utilization and improvements in the population’s health status, including better control of the three diseases [40].

A different experience occurred in Namibia, a lower middle-income country in sub-Saharan Africa. Namibia received an influx of donor funding between 2004 and 2007 to support publicly provided HIV care and treatment. This raised concerns that private funding would be “crowded out,” thereby leading to a reduction in the overall resources used to treat patients. In 2006, the Namibian medical aid industry, with donor support, created a special fund to subsidize private health insurance, including HIV services. The program allowed both low- and higher-income people to be covered. Crowding out valuable private resources was avoided and the quality of HIV services improved [41].

Social health insurance (SHI) schemes are based on mandatory enrollment, which maximizes risk pooling and guards against adverse selection. Several of those programs provide full coverage for HIV and operate successfully in upper middle-income and high-income countries. In countries with low HIV prevalence, a large enough portion of the population has formal employment and can therefore contribute payroll-based premiums. Argentina, Chile, Colombia, El Salvador, Mexico, Paraguay, Peru, and Uruguay have such systems. Based on UNGASS data from 2010, 2012, and 2013, those schemes provide 60% of the HIV funding Chile (high-income), and 69% in Colombia (upper middle-income), but the proportion is substantially lower in El Salvador and Paraguay (both lower middle-income), Peru (upper middle-income), and Uruguay (high-income), where those schemes cover 8% to 11% of the national HIV funding.

Chile’s AUGE health reform, enacted in 2005, mandates public and private health insurers to cover treatment for 69 priority health problems, including HIV. Even when the HIV prevalence is low and a large number of people are enrolled in the scheme, additional funding beyond the premiums might be required, as is the case in Brazil’s *Sistema Unico de Saûde,* which fully covers HIV prevention and treatment, and is financed by a combination of payroll and general tax revenues.

Another type of risk-pooling scheme is special social assistance programs to benefit people living with a particular illness, including HIV. For instance, in Jamaica, the government operates two individual drug benefit programs under the National Health Fund (NHF) that cover citizens for a number of chronic illnesses and conditions including arthritis, diabetes, hypertension, vascular disease, and high cholesterol. Members enrolled in these programs are eligible to receive 300 subsidized drugs from private and public pharmacies, which, in turn, are reimbursed by the NHF. Members are not required to pay a premium, but they do need to make the appropriate copayments. NHF is financed from partial allocations from the special consumption tax revenues. Lately, the NHF drug benefit programs are being used as a mechanism through which people living with HIV can access free antiretroviral drugs [42].

If well designed and implemented, health insurance can prevent catastrophic health expenditures and, consequently, impoverishment. Health insurance can encourage prevention, early diagnosis, and treatment by removing the financial barriers to access these services. As shown above, in several middle- or upper middle-income countries, health insurance schemes are able to cover HIV services, at least partially. In many low- and middle-income countries, however, public and private health insurance is underdeveloped. There are two major reasons for this. First, in many of those countries a large share of the labor force is informal, which makes mandatory enrollment and premium collection difficult. Second, many public institutions are weak and they may be unable to regulate health insurance to avoid the typical problems of insurance such as adverse selection, beneficiary exclusion by the insurer, cost escalation, misinformation, and deficient quality of care.

### Kenya case study

Decreasing donor dependence is a challenge as reflected in Kenya, where several options for replacing the diminishing donor HIV funding were laid out in 2009, including two of the above options discussed above: establishing a small levy on airline traffic (with negligible impact on air travel); increasing the government’s budget allocation to health to 15% in order to achieve the Abuja targets (assuming the percentage of the health budget allocated to HIV remains constant); and modestly raising premiums in the National Hospital Insurance Fund to generate sufficient financing for the scheme to cover essential HIV services. If all three financing options were to be implemented, the HIV donor funding dependency would decrease from 87% to 68%, still a very high dependence on donor funding. Getting those options approved is a challenge on its own, though in 2012, after three years of lengthy negotiations with the Ministry of Finance, the Cabinet approved the Bill entitled Additional and Sustainable Financing for HIV/AIDS and Non-Communicable Diseases in Kenya [8]. The government of Kenya planned to earmark 1 per cent of its revenue to a national HIV trust fund, which on its own covers 74% of the AIDS financing gap in the ten years up to 2020.

## Discussion and conclusion

Four non-donor-based funding sources for HIV programs were identified: earmarked levies for HIV; concessionary loans; debt conversion; and risk-pooling schemes. Three of them are domestic and one is external (debt conversion). In three, the funding agent is the Ministry of Finance; the fourth uses health insurance entities. In both Kenya and Zimbabwe, earmarked levies for HIV would generate or have generated, respectively, only 15% of the annual HIV response cost; in Indonesia, debt conversation was also only 15% of the estimated cost of the HIV response. While the €20 million debt conversion in Pakistan represents 45% of the annual cost of Pakistan’s response to its low-level HIV epidemic, this sum would have covered a smaller share of the cost of HIV responses in more generalized epidemics. In addition, debt conversion is a one-off mechanism rather than an ongoing funding source. Likewise, concessionary loans are not a recurring source of funding, they require a payment that low-income countries might find hard to cover, and they may not produce the large amount of funding required to fight HIV. Given the high-cost of HIV-related services, in particular of ART, the applicability of health insurance, which is solely supported by individual contributions, is influenced by a range of factors, including the income level of the country, the severity of the HIV epidemic, the proportion of the labor force from which premiums can be collected, and the ability of the country to regulate health insurance.

Understandably, we are not aware of every low- and middle-income country that uses the funding mechanisms mentioned here. Furthermore, some dimensions such as transaction costs and social acceptability were not compared due to lack of data. When explaining the magnitude of some of the mechanisms, we used the cost of the HIV response, as reflected in funding requests submitted to the Global Fund. Although these requests might contain overestimations, intended to help the requesting country secure more funding, it is those estimates that are used to justify resource mobilization.

The above analysis indicates that not all alternatives described in this paper will fit every country. Also, no one solution will provide for the entire funding needs of a given HIV response, at least not in the medium and long term. Solutions should be context specific, factoring in income and epidemic levels as well as other things. With no magic bullet to lessen the decline in HIV donor funding, HIV programs should always first aim to reduce cost by becoming more efficient and prioritizing services, not solely aim at mobilizing additional resources. If a non-donor funding source is to be explored, countries should consider not only the expected revenue, but also the transaction costs, the ability to implement the mechanism with existing infrastructure, the transparency of financial flows and accountability and ability to prevent fraud or misuse of funding, social acceptance and distribution of financial burden, and benefits across sub-populations within the country.

In the process of mobilizing additional financial resources, there is a need to involve the Ministry of Finance from the very beginning, as shown in the Kenya case study. Ministries of Finance are the stewards of the financial equilibrium of the country and the response to the epidemic, or the lack of it, will frequently impact this equilibrium. The strategies presented in this paper would require Ministry of Finance involvement and support, both technical and political. This support can be acquired by developing with them a costed HIV strategy that will include cost reductions (technical efficiency) and outcome improvements (allocative efficiency), efficiency measures that will justify the investment in such strategy. The discussion with the Ministry of Finance can be supported with evidence on the savings gained from investments in preventive health care. Managed care for chronically ill patients yields net economic benefits by reducing future treatment costs in areas such as hospitalizations, surgeries, and other complications, as is the case with disease management for diabetic and hypertensive patients (e.g., Beaulieu et al. [43], Bradbury [44], and Sidorov et al. [45]). An important article by Resch et al. [46] argues that public investments in ART result in net positive economic benefits. The authors estimated the cost of maintaining ART during a 10-year period (2011–2020), for the 3.5 million patients in low- and middle-income countries worldwide. Next they estimated the economic savings resulting from such treatment. The authors concluded that the investment required for ART, equal in present value to US$14.2 billion, is expected to save 18.5 million life-years and return US$12–34 billion through increased labor productivity, averted orphan care, and deferred medical treatment for opportunistic infections and end-of-life care. According to the Global Fund, investing $120 million a year on AIDS treatment in Namibia frees up 9,200 hospital beds for other patients each year and spares the lives of 1,000 health workers and 550 teachers. During a five-year period, Namibia has seen AIDS deaths drop from 2,700 annually to 56 [47]. In addition to the economic return, the Ministry of Finance needs to recognize that increasing domestic funding can retain or attract external funding, as increasingly donors require counter-funding, as discussed above.

The lengthy and complex process of mobilizing non-donor funding suggests both countries and donors would benefit from formulating a financial sustainability plan before any donor funding for HIV programs and activities is committed, to ensure the funded activities also continue after the donor funding declines or ends. The sustainability plan should include specific actions as to how the recipient country or organization will gradually replace donor funds with domestic and/or internal financing over a specific timeframe. This timeframe might vary across countries, by income level and by burden of HIV. In the face of their likely ineligibility for certain donor funding, it is arguably critical that upper middle-income countries, especially those with high burden of HIV, undertake immediate measures for replacing donor funds with domestic resources and other alternative funds over a relatively shorter timeframe.
